# The Immune and Non-Immune Pathways That Drive Chronic Gastrointestinal Helminth Burdens in the Wild

**DOI:** 10.3389/fimmu.2018.00056

**Published:** 2018-02-05

**Authors:** Simon A. Babayan, Wei Liu, Graham Hamilton, Elizabeth Kilbride, Evelyn C. Rynkiewicz, Melanie Clerc, Amy B. Pedersen

**Affiliations:** ^1^Institute of Biodiversity, Animal Health & Comparative Medicine, University of Glasgow, Glasgow, United Kingdom; ^2^Glasgow Polyomics, Glasgow, United Kingdom; ^3^Institute of Evolutionary Biology and Centre for Immunity, Infection and Evolution, School of Biological Sciences, University of Edinburgh, Edinburgh, United Kingdom

**Keywords:** wild immunology, *Apodemus sylvaticus*, transcriptome, machine learning applied to immunology, *Heligmosomoides polygyrus*, anthelminthics

## Abstract

Parasitic helminths are extremely resilient in their ability to maintain chronic infection burdens despite (or maybe because of) their hosts’ immune response. Explaining how parasites maintain these lifelong infections, identifying the protective immune mechanisms that regulate helminth infection burdens, and designing prophylactics and therapeutics that combat helminth infection, while preserving host health requires a far better understanding of how the immune system functions in natural habitats than we have at present. It is, therefore, necessary to complement mechanistic laboratory-based studies with studies on wild populations and their natural parasite communities. Unfortunately, the relative paucity of immunological tools for non-model species has held these types of studies back. Thankfully, recent progress in high-throughput ‘omics platforms provide powerful and increasingly practical means for immunologists to move beyond traditional lab-based model organisms. Yet, assigning both metabolic and immune function to genes, transcripts, and proteins in novel species and assessing how they interact with other physiological and environmental factors requires identifying quantitative relationships between their expression and infection. Here, we used supervised machine learning to identify gene networks robustly associated with burdens of the gastrointestinal nematode *Heligmosomoides polygyrus* in its natural host, the wild wood mice *Apodemus sylvaticus*. Across 34 mice spanning two wild populations and across two different seasons, we found 17,639 transcripts that clustered in 131 weighted gene networks. These clusters robustly predicted *H. polygyrus* burden and included well-known effector and regulatory immune genes, but also revealed a number of genes associated with the maintenance of tissue homeostasis and hematopoiesis that have so far received little attention. We then tested the effect of experimentally reducing helminth burdens through drug treatment on those putatively protective immune factors. Despite the near elimination of *H. polygyrus* worms, the treatment had surprisingly little effect on gene expression. Taken together, these results suggest that hosts balance tissue homeostasis and protective immunity, resulting in relatively stable immune and, consequently, parasitological profiles. In the future, applying our approach to larger numbers of samples from additional populations will help further increase our ability to detect the immune pathways that determine chronic gastrointestinal helminth burdens in the wild.

## Introduction

Chronic helminth infections challenge our understanding of how the immune system functions. In natural populations, while individuals are continually exposed to helminth parasites, there is substantial variability in infection burdens, with some consistently showing no active infection (1, 2). This suggests that individuals can potentially control infection. In addition, while anthelminthics are commonly employed to reduce helminth burdens, after drug clearance, infections typically return to their initial burdens (3–5), suggesting that the immune system does not or cannot easily acquire complete protection against helminths. Such observations have lead to the hypothesis that hosts must balance the costs of helminth infection with the immunopathology and/or protein-energy-related costs associated with eliminating these parasites (6–8). If this trade-off exists, it suggests that the host can regulate the intensity of its immune attack on the parasites, and, alternatively, that these parasites can avoid and suppress host immune responses (9, 10). While these ideas have received substantial theoretical and experimental support, the mechanisms that underlie chronic helminth infection dynamics “in the real world,” and whether or how the balance of resistance and susceptibility might change over the lifetime of an individual, either naturally or in response to vaccination, remain very poorly understood. We suggest that addressing these questions is necessary for the development of more effective and sustainable treatment and immunization against parasitic helminths, which are the leading cause of productivity loss in livestock (11–13) and remain a substantial agent of poverty in the developing world (14, 15). It is, therefore, paramount to study hosts in their natural environment, outside of the controlled laboratory, where the data may be messy, but where relationships between hosts and their natural parasite communities are the result of millions of years of coevolution and more likely to be biomedically relevant.

Identifying protective immune mechanisms in inherently variable natural populations warrants relatively large sample sizes, the possibility to manipulate parasitological, physiological, and environmental parameters, and the ability to monitor individuals repeatedly over time. The wood mouse *Apodemus sylvaticus* has been extensively studied by disease biologists since the 1960s (16, 17) and is now increasingly used for immunological studies (18, 19). It offers many of the features that originally made small rodents attractive experimental models (i.e., affordable, easy to maintain and handle, prolific breeding, and short time to maturity) and adds features that make it an ecologically and epidemiologically sound model for mammalian, including human, immunology, and parasitology (i.e., high diversity and prevalence of parasites, good trapability, large population sizes, genetic relatedness to *Mus musculus*). Indeed, *A. sylvaticus* harbors a diverse and prevalent community of parasite and pathogens that closely resembles those found in larger mammals, including humans (20–24), and domestic animals (25). Importantly, unlike *Mus musculus* which is naturally infected by very few gastrointestinal helminth parasites (26, 27), *A. sylvaticus* is the natural host of the nematode *Heligmosomoides polygyrus*, which is routinely found in >50% of wild wood mice (28) and is closely related to *H. bakeri* (29), a species extensively studied as a model of human gastrointestinal helminths that is known for its ability to suppress the immune system of its host (30). In this study, we therefore used *H. polygyrus* as a model for chronic endemic helminthiasis, and aimed to identify immune networks that regulate infection burden in a natural host–helminth system. Due to the preponderance of the laboratory mouse model in immunology, there are few, if any, reagents developed and optimized for non-model organisms (31). We, therefore, utilized a transcriptomics approach. While the genome itself provides invaluable information about immune resistance to infection (32–34), messenger RNA expression profiles provide a time- and context-sensitive picture of the immune system as it responds to antigenic stimuli. Moreover, unlike PCR and other candidate gene-driven approaches, transcriptomics allow discovery of unexpected correlates of immune protection and inclusion of physiological processes in the discovery of determinants of resistance to disease that would otherwise be overlooked in a purely immunology-focused approach.

In this study, we aimed (i) to identify gene networks that were either positively or negatively associated with *H. polygyrus* infection burden, (ii) to use those genes to identify immune pathways that promoted immunity to *H. polygyrus*, and (iii) to test whether those protective pathways were affected by sex and anthelminthic drug treatment. We used a newly published *A. sylvaticus* genome for the assembly and analysis of RNAseq samples generated from the spleens of 34 wild-caught wood mice from two distinct populations showing natural variation in helminth burdens, and then randomly treated a subset with anthelminthics to experimentally reduce their nematode infection burdens. Identifying transcripts predictive of infection burdens in a natural system, and maximizing the chances of these associations to generalize across populations requires detecting potentially weak signals among many variables, while simultaneously avoiding spurious variation (false positives). We addressed this challenge by reducing transcripts into co-expression gene networks, and identifying therein robust predictors of immunity to *H. polygyrus* using supervised machine learning—a class of statistical models that can integrate multiple variables that each carry weak signal into a stronger predictor by mapping them to a response variable, here *H. polygyrus* burdens. These types of supervised machine learning approaches hold promise for ecological and immunological studies (35, 36). We then applied a similar supervised approach to a narrower set of transcripts explicitly associated with immunity to assess how the immune system regulates its response to chronic helminth burdens in both male and female mice. To ensure that our models were not specific to a single population, time point, and sequencing platform, and thereby to increase the generalizability of our conclusions, we split samples spanning two wood mouse populations repeatedly at random into training sets on which predictive models were generated, and corresponding test sets on which the predictions of trained models were validated against observed values. Finally, given the widely reported propensity of anthelminthic-treated individuals (humans included) to return to their initial helminth burdens within weeks, we assessed whether the correlates of protection identified by our supervised learning approach were impacted by anthelminthic treatment.

## Materials and Methods

### Ethics Statement

All procedures on animals were approved by the University of Glasgow ethics committee and the UK Home Office (PPL60/4572) and conducted in accordance with the Animals (Scientific Procedures) Act 1986.

### Wood Mouse Field Treatment and Sampling

To minimize false discovery and ensure that our findings could generalize to other wood mouse populations, we included samples from two geographically and temporally distinct *A. sylvaticus* populations at two woodland sites: Haddon Wood (N 53.16°, W 3.1°, hereafter referred to as HW) in north-west England in 2011, and Callendar Wood (N 55.99°, W 3.78° hereafter referred to as CW) in the Central Lowlands of Scotland in 2015.

Mice were trapped using Sherman live traps (H. B. Sherman 2 × 2.5 × 6.5 inch folding trap, Tallahassee, FL, USA) baited with grain, carrot, and bedding material. Two traps were placed every 10 m in a 70 m × 70 m (total 128 traps per grid). At first capture, each individual was microchipped (AVID microchips, Lewes, UK). In CW only, mice were allocated to one of two sex-balanced groups: anthelminthic drug-treated or control. Age and reproductive status, assessed by the body mass, color of the coat, position of testes, occlusion of the vagina, and visible signs of lactation and pregnancy were also recorded. The drug treatment removed gastrointestinal nematodes, which are typically the most abundant parasites in wood mice (4). Drugs were given as a single oral dose of 2 µl g^−1^ of body weight of a mixture of 9.4 mg kg^−1^ Ivermectin and 100 mg kg^−1^ Pyrantel (IVM + PYR), and controls consisted of oral delivery of 2 µl g^−1^ of body weight of water (H_2_O). Mice that were recaptured 14 ± 3 days post first capture were sacrificed on site. After cervical dislocation and exsanguination by cardiac puncture, the spleen of each individual was extracted and immediately transferred to a tube containing 4 ml RNA later, followed by whole removal of the intestine and storage in phosphate-buffered saline for subsequent dissection and parasite identification.

### Parasitology

*Heligmosomoides polygyrus* are ingested at their third larval (L3) stage, penetrate the submucosa of the small intestine within 24 h, and migrate to the muscularis externa, where they develop into L4-stage larvae. 8–10 days after infection, adult worms begin to emerge into the lumen of the intestine and attach to the intestinal villi within ~14 days, where they mate and release eggs. We measured adult *H. polygyrus* intestinal burdens of all sacrificed mice to assess immune resistance to infection. Worm burdens are preferred over fecal egg counts because they have much lower variability and likely reflect interactions with the host’s immune system during larval development. While it was not possible to measure individual level differences in exposure to parasite infective L3s in the wild, we selected mice of similar ages within both sexes, and assumed lifetime exposure levels were similar across all selected mice. Worm burdens were compared between sites (CW or HW), sexes and treatment groups using negative binomial generalized linear models because of the significant left-skew coupled with high worm prevalence, over a zero-inflated distribution.

### RNA Sequencing, Assembly, Annotation, and Quantification

A transversal segment of each spleen was cut under sterile conditions, weighed, and processed following the RNeasy kit (Qiagen). RNA quantity and quality were assessed using a Tapestation (Agilent Technologies), and stored at −80 until RNA sequencing. All RNA samples were evaluated on a Bioanlayzer (Agilent Technologies) immediately prior to Poly-A selection of messenger RNA. Ten wood mice from Haddon Wood were sequenced on an Illumina Solexa 454 with 100 bp paired end reads by Edinburgh Genomics (2011). Twenty-four wood mice from Callendar wood were sequenced on an Illumina NextSeq 500 with 75 pb paired end reads by Glasgow Polyomics (2015). Raw reads generated by the sequencers were quality-checked using FastQC v. 0.11.5 (37) and sequences of low-quality and sequencer adapters trimmed using cutadapt v. 1.14 (38). The resulting trimmed reads were then quality-checked using FastQC and MultiQC (39) as above (see MultiQC report in Supplementary Data Sheet 1), and aligned to a reference transcriptome and quantified using Kallisto v. 0.43.1 (40). An *A. sylvaticus* transcriptome generated from a recently published genome (assembly ASM130590v1, https://www.ncbi.nlm.nih.gov/nuccore/LIPJ00000000.1) was used as the reference for assembly and read counts.

### Transcriptome Analysis

#### Dimensionality Reduction

To select within the full transcriptome only the genes that had a potential role in resistance or tolerance to *H. polygyrus* across the full wood mouse transcriptome, we first grouped highly interconnected transcripts that may form distinct biological pathways by applying a weighted correlation network analysis (41) with the R package WGCNA (42). This entailed constructing gene networks (or modules) using a co-expression similarity measure defined as:

$${\text{A}}_{\text{ij}}={\left(0.5+0.5{\text{cor(x}}_{\text{i}}{\text{, x}}_{\text{j}}\text{)}\right)}^{\beta }$$

where A_ij_ is the pairwise correlation between gene expressions (x_i_, x_j_), and β is the soft-threshold weight which is set at 6 in our analysis based on scale-free topology criterion (41). The WGCNA cluster eigengenes, which summarize the expression levels of all transcripts within each cluster, were used for further analysis as combining such clusters into biologically linked “meta-networks” as been shown to help identify biologically meaningful pathways (43–45). In addition to identifying the strongest correlates of protection across the full transcriptome, we sought to describe how genes explicitly associated with immunity might be functionally associated with *H. polygyrus* infection burdens. We, therefore, selected all genes for which the BLAST annotation contained the following immunological terms: “chemokine,” “cytokine,” “gata3,” “immunoglobulin,” “interferon,” “interleukin,” “platelet,” “relm,” “resistin,” “ror-gamma,” “t-bet,” “TBX21,” “TGF,” “TNF,” or “toll-like” for their reported roles in immunity or regulation of responses to parasitic nematodes (9, 46–49).

#### Supervised Learning

To identify WGCNA clusters or immune genes (“features”) that may have a role in regulating *H. polygyrus* burdens, we trained supervised learning models to map them to parasite counts of untreated individuals from both CW and HW populations. All features were scaled to unit variance to ensure homoscedasticity, and worm counts were log10+1-transformed to improve machine learning algorithm performance. To predict *H. polygyrus* burdens, we used a regression analysis with the task of minimizing mean squared errors (MSE) between parasitic worm burdens observed in a test set and burdens predicted by a model trained on an independent training subset of the full dataset.

To select which algorithm was most likely to generate the best models from our data, we began by comparing the baseline performance of widely used algorithms using their default settings on the full dataset using 10-fold cross-validation: Elastic Net; k-Nearest Neighbors for Regression; Random Forest regressor; and eXtreme Gradient Boosting regressor using either a linear (GLM) booster, or a tree booster.

The full dataset was then split 75/25 into a training set and the corresponding test set 10 times repeatedly at random without replacement to avoid sampling bias affecting our choice of trained model. For each of the random train-test paired subsets, the selected algorithm was tuned on the training set using a wide range of possible parameter settings on the training subset using fivefold cross-validation, and the trained models that achieved the lowest MSE between predicted and observed burdens in the test set were retained. Summary statistics (mean and SEM) of all 50 resulting MSE, and of all 50 weights applied to each cluster within the trained models were used for ranking the importance of each gene transcript or cluster in predicting *H. polygyrus* burdens.

#### Statistical Analysis

Immune features (WGCNA clusters and transcripts) that contributed most to the predictive performance of each model were used as response variables in generalized linear models to assess the effects of host sex (two level factor) and anthelminthic treatment (two level factor) on their expression. To reduce type 1 errors due to multiple testing, statistical significant was considered at *p* ≤ 0.01.

All data processing, machine learning, statistical analyses, and graphs were performed using Python 3.6 packages pandas (50), scikit-learn (51), statsmodels (52), and seaborn (53), respectively.

## Results

### Infection by *H. polygyrus* Was Prevalent but Infection Intensity Varied Substantially between Individuals and Populations

**Figure 1:**
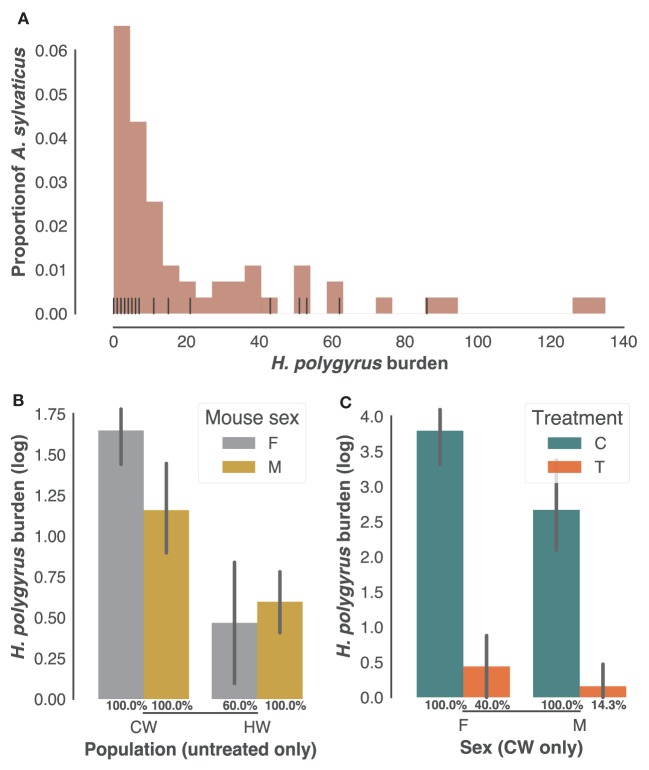
*Heligmosomoides polygyrus* prevalence and burdens in sampled *Apodemus sylvaticus*. **(A)** Distribution of *H. polygyrus* intestinal burdens in 61 *A. sylvaticus* sampled in HW and CW (histogram) and among those the 34 individuals randomly selected for the transcriptome analysis (vertical black lines). **(B)** *H. polygyrus* burdens of untreated male (M) and female (F) mice from CW and HW. **(C)** *H. polygyrus* burdens of treated (T) and untreated controls (C) from CW only. In **(B,C)** *H. polygyrus* prevalences in each category are given as percentages, bars represent mean log-transformed worm counts and error bars show the SEM.

As is typical of parasite burdens where a minority of the animals carry most of the infection (54), *H. polygyrus* infection burdens followed a negative binomial distribution in the 61 individuals sampled in the two field populations (CW and HW), with burdens ranging from 0 to 135 worms (Figure 1A). Of those, 34 adult mice selected at random with *H. polygyrus* burdens at post mortem ranging 0–86 were retained for further analysis. While worm burdens within each population did not to differ significantly between sexes (Figure 1B), they were markedly higher in CW than in HW (Figure 1C, *p* < 0.0001, *n* = 22 untreated wood mice). All males and all females from CW included in subsequent analyses were infected with *H. polygyrus*, while prevalence was 60% for the females from HW (Figure 1B), amounting to a 91% prevalence of *H. polygyrus* in our untreated individuals. Half of the males and females caught in CW were treated with anthelminthic drugs to test associations between transcript expression (Illumina RNA-seq counts) and gastrointestinal helminth burdens. Treatment was equally effective at reducing *H. polygyrus* numbers and prevalence in both sexes but failed to remove the parasites completely (Figure 1C, 60× reduction in burden *p* < 0.0001; prevalence *X*^2^ = 0.0007, *n* = 24 in CW only). Taken together, these results confirm that this parasite is common in *A. sylvaticus*, suggest that *H. polygyrus* varies substantially in intensity between and within populations, and that anthelminthic treatment, while effective, did not completely eliminate gastrointestinal helminths from the treated individuals.

### Transcriptome-Wide Correlates of Resistance and Susceptibility to *H. polygyrus* Were Highly Predictive of Worm Burden

**Figure 2:**
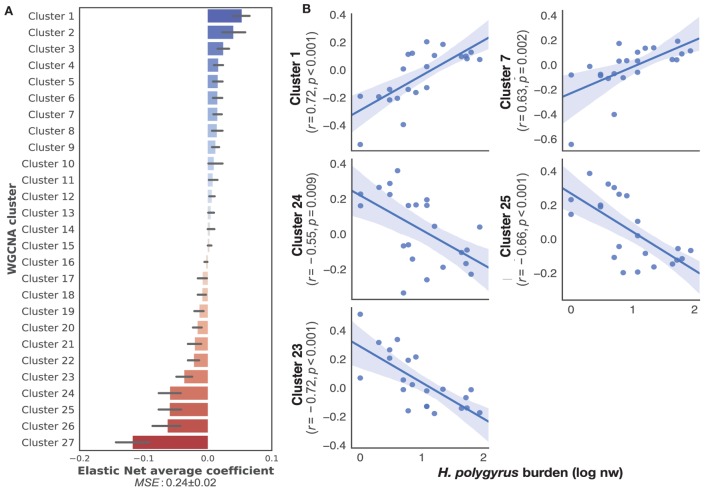
Top gene networks predictive of chronic *Heligmosomoides polygyrus* worm burdens in wild wood mice. **(A)** Average ranking of WGCNA clusters based on Elastic Net coefficients of 50 models trained on 22 untreated wood mice from HW and CW (see Materials and Methods for description details). **(B)** Regression plots of the top five positive and negative clusters based on their coefficients against log-transformed *H. polygyrus* burdens, for which *p* ≤ 0.01. Each point represents a wood mouse, the solid line is the regression line and the shaded areas represent the corresponding 95% bootstrapped confidence intervals. KEGG pathways (human and mouse) associated with: Cluster 1: ubiquitin protein ligase synthesis and terpenoid backbone synthesis; Cluster 7: ATP-dependant RNA helicase activity, NOD-like receptor signaling pathway; Cluster 24: phosphatidylinositol 3-kinase regulatory subunit binding, glucosidase activity, apoptosis; Cluster 25: C2H2 zinc finger domain binding, disordered domain specific binding; Cluster 23: protein tyrosine kinase activity, ATP-binding cassette transporters.

To identify transcriptome-wide gene expression profiles that might explain the observed variation in worm burdens and help identify immune mechanisms that regulate *H. polygyrus* burdens in wild wood mice, we began by clustering transcripts into co-expression networks to reduce the number of variables for further analysis. WGCNA reduced the 17,639 transcripts contained in the full transcriptome to 131 clusters. We used the eigengene (or first principle component) of each cluster to capture the majority of variation of all the genes contained within that cluster. Despite differences in infection burdens reported above, we found no clustering of transcription profiles by sampling origin, sex, or treatment (Figure S2 in Supplementary Material), indicating that our sampling procedure, use of different sequencing platforms, host sex, and drug treatment did not cause transcriptome-wide biases between individuals. All 34 transcriptomes were, thus, treated as belonging to the same statistical population. To identify the clusters associated with chronic *H. polygyrus* burdens, we used a supervised learning approach with clusters entered as explanatory variables and log10+1-transformed *H. polygyrus* counts as the response variable. Only mice that were not treated with anthelminthics were considered. A comparison of machine learning algorithms using fivefold cross-validation suggested two classes of algorithms were best suited for generating models mapping gene expression clusters to *H. polygyrus* burdens, gradient boosting with a linear booster (55) and Elastic Nets (Figure S3 in Supplementary Material). Elastic Nets identified several positively and negatively correlated clusters with robust statistical support. Elastic nets are particularly useful when the number of predictors is larger than the number of observations, as they tend to group together highly correlated features, a desirable behavior when selecting gene expression variables (56). Conversely, the XGBoost algorithm selected few clusters with clear positive of negative associations with worm burdens and appeared too sensitive to outliers, suggesting that it was likely to overfit (Figure S4 in Supplementary Material)—we, therefore, retained only Elastic Nets for further analysis. We then trained Elastic Nets as described in the section “Materials and Methods” on 10 randomly selected training subsets of untreated only mice from both CW and HW. The resulting models predicted log10+1-transformed *H. polygyrus* burdens in the corresponding test sets with a MSE of 0.24 ± 0.01. To identify clusters that were most informative to the prediction models, we ranked them using the Elastic Net coefficients, of which both positively- and negatively associated transcript expression networks were identified (Figure 2A). Within the 10 top-ranking clusters, we only retained for further analysis the five clusters that correlated significantly with worm burden at *p* ≤ 0.01 (Figure 2B). The KEGG pathways associated with the gene transcripts that correlated positively with parasite burdens included farnesylated proteins-converting enzyme 1, ubiquitin protein ligase synthesis and terpenoid backbone synthesis, ATP-dependant RNA helicase activity, and the NOD-like receptor signaling pathway (see Table S1 in Supplementary Material for details of all clusters positively associated with worm burdens). Pathways negatively correlated with parasite burdens included apoptosis, phosphatidylinositol 3-kinase regulatory subunit binding, glucosidase activity, C2H2 zinc finger domain binding, disordered domain specific binding, protein tyrosine kinase activity, and ATP-binding cassette transporters (see Table S2 in Supplementary Material for details of all clusters negatively associated with worm burdens). Host sex did not significantly affect the expression of either sets of genes (Figure S5 in Supplementary Material).

### Immune Gene Transcription Correlates of Resistance and Susceptibility to *H. polygyrus*

**Figure 3:**
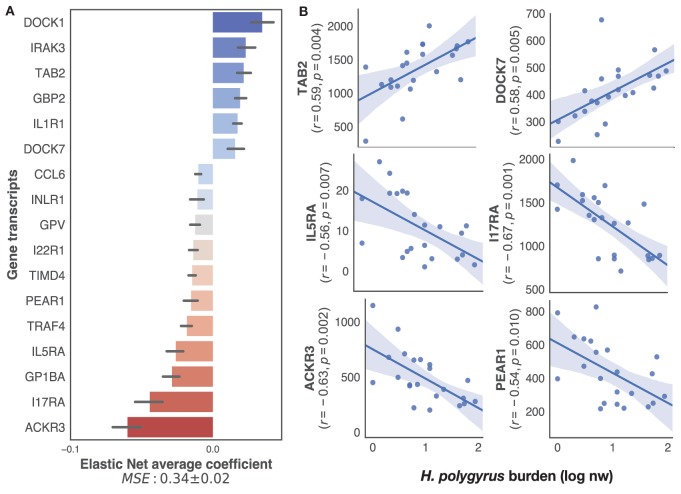
Top immune predictors of chronic *Heligmosomoides polygyrus* worm burdens in wild wood mice. **(A)** Average ranking of immune gene transcripts based on Elastic Net coefficients of 50 models trained on 22 untreated wood mice from HW and CW (see Materials and Methods for details on immune gene selection procedure and model training). **(B)** Regression plots of the top five positive and negative clusters based on their coefficients against log-transformed *H. polygyrus* burdens, for which *p* ≤ 0.01. In regression plots, each point represents a wood mouse, the solid line is the regression line and the shaded areas represent the corresponding 95% bootstrapped confidence intervals.

To specifically identify, within the transcriptome, only immune genes associated with the regulation of *H. polygyrus* burdens, we applied our supervised learning approach to transcripts selected on the basis of their BLAST annotations containing explicit reference to immune function (see Materials and Methods for details). This retained a list of 222 unique genes out of the 12,437 present in the full transcriptome. Elastic nets mapping those immune transcripts to log10+1-transformed *H. polygyrus* burdens predicted *H. polygyrus* burdens of mice in the test datasets with a MSE of 0.33 ± 0.02 (Figure 3A). Among the transcripts that contributed most to the prediction, eight were significantly (*p* ≤ 0.01) correlated with parasite burden (Figure 3B), 2 positively—which included TGF-β-activated kinase 1 and MAP3K7-binding protein 2 (TAB 2), and dedicator of cytokinesis protein 7 (DOCK7), and 4 negatively—which included interleukin-5 receptor alpha, interleukin-17 receptor alpha, atypical chemokine receptor 3 (ACKR3), and platelet endothelial aggregation receptor 1.

### Effect of Sex and Treatment on Immune Pathways Correlated with Parasite Burdens

**Figure 4:**
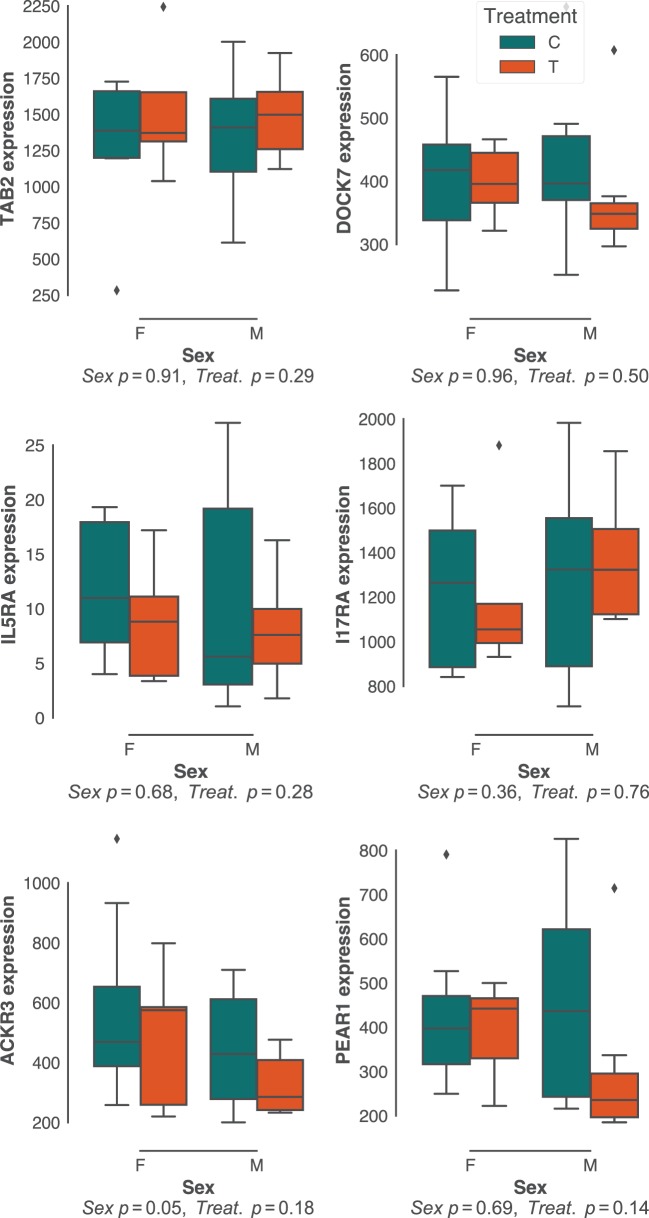
Effects of host sex and drug treatment on protective immune gene expression. No statistically significant effects of host sex and drug treatment were detected among the best predictors of *Heligmosomoides polygyrus* burdens identified in untreated mice only. Each plot represents transcript counts scaled to unit variance but not centered. Horizontal bars represent the median, boxes represent the interquartile range, whiskers the range, and diamonds transcript counts that lay beyond 1.5× of the interquartile range.

Sex effects are widely reported to affect gastrointestinal parasite burdens (57–60). Although there was little sex bias in *H. polygyrus* burdens in our study population (Figure 1B; Figure S1B in Supplementary Material), we did investigate whether the expression of protective pathways identified above differed between sexes. In addition, we predicted that experimental reduction of parasite burdens through anthelminthic treatment, which had resulted in a dramatic reduction in *H. polygyrus* worm burdens (Figure 1C), would profoundly affect the expression of immune genes associated with responses to these parasites. Contrary to our expectations, neither sex nor drug treatment resulted in major differences in expression of gene networks (Figure S2 in Supplementary Material), nor specifically in the expression of genes that correlated with *H. polygyrus* burdens in untreated individuals (Figure 4). Because our choice of Elastic Nets favored linear relationships and may, thus, have failed to identify non-linear relationships between gene expression and worm counts, we repeated the analyses above using gradient boosted trees. Consistent with expectations (Figure S3 in Supplementary Material), XGBoost achieved very similar predictive performances to the Elastic Nets (mean MSE [range] = 0.34 [0.11–3.3], Figure S6 in Supplementary Material), and of the top features, two out of the four that were significantly correlated with parasite burden were in agreement with those identified by the Elastic Nets (DOCK7 and IL17RA, Figure S6B in Supplementary Material). The two other genes identified by XGBoost were suppressor of cytokine signaling 2 (SOCS2) and interferon-stimulated 20 kDa exonuclease-like 2 (I20L2), which both showed greater variance in gene expression at intermediate parasite burdens. SOCS2 and I20L2 were expressed at only marginally different levels between treated and untreated animals (*p* = 0.04 and *p* = 0.05, respectively), and between sexes (*p* = 0.15 and *p* = 0.03, respectively) (Figure S7 in Supplementary Material).

## Discussion

The distribution of parasite infection burdens within a population is typically highly dispersed, with a small proportion of the hosts harboring the heaviest infections (61). Here, the prevalence of *H. polygyrus* was high in both populations studied, and remained detectable even after drug treatment. This suggests, assuming similar exposure to infection at a given age, that most individuals limited their parasite burdens but did not eliminate infection completely, consistent with reports on chronic helminth infections across diverse host species (61–63). Furthermore, drug treatment usually needs to be administered regularly to control helminth infections, as otherwise worms recrudesce within weeks (3, 5, 64). We sought to better understand how the immune system, under natural exposure and chronic helminth infection, regulates its response to infection, what immune traits allow some hosts to maintain low infection burdens, and how anthelminthic treatment and the subsequent reduction in parasite burdens impact those protective immune traits.

Addressing these questions required choosing a species that naturally harbors a diverse community of parasites at high prevalence and densities to maximize our chance of detecting the effects of their removal on the host. This ruled out *M. musculus*, which despite being a powerful model for immunology, is a relatively poor model for parasitology owing to the uncharacteristically low helminth infections naturally present in that species (26, 27). Wood mice, however, resemble species of greater societal importance in the diversity and prevalence of the parasite communities (21, 65). Here, we chose to focus on the gastrointestinal nematode *H. polygyrus* due to its prevalence in wood mice, and to its extensive use as a laboratory model (24, 66, 67), thus providing a powerful way to compare lab-based and field-based immune responses to this parasite. In our study populations, *H. polygyrus* was present in 91% of the wood mice we caught.

The immune system of *A. sylvaticus* is poorly known, thus we chose to take a transcriptomic approach to discover specific host–parasite interactions. In addition, having the full transcriptome also allowed us to use a candidate gene approach based on BLAST annotations. Moreover, it is likely that a significant amount of variation in chronic helminth burdens could be driven by processes not typically classified as immunological. We, therefore, decided to combine the discovery approach afforded by our sequencing of the full transcriptome with an approach focused on the genes explicitly associated with the immune system. However, RNAseq risks overestimating transcript abundance due to splicing variants, or being dominated by the more abundantly transcribed genes. While a technical solution would be to combine platforms with different read depths and lengths, here we used analytical solutions (e.g., FastQC reports of overrepresented sequences), and supervised learning to focus on transcripts that had statistically significant correlation with a biological read-out, here, parasite burdens. In short, we used parasite burdens to select the genes and co-expression networks for further analysis. By using Elastic Nets to identify correlates of parasite burdens, we ensured that redundant transcripts would feature together in the ranking of coefficients once models were trained. Yet, after removing all exact duplicates from the raw transcript counts, our models generated lists of uniquely represented genes, suggesting that for any that may have had differently expressed isoforms, only one of each correlated significantly with parasite burden.

We found that within the transcriptome, the best predictors of parasite burdens included both immune and non-immune genes. Among non-immune genes that were associated with *H. polygyrus*, were farnesylated proteins-converting enzyme 1 (FACE1), which is involved in protein hydrolysis (68), dedicator of cytokinesis protein 7 (DOCK7), which is reported to influence lipid regulation (69, 70), and proteases, helicases, and ABC transporters—genes generally involved in protein and energy metabolism, and potentially tissue growth and/or repair. Although nutrition plays an important role in resistance to helminth infections (71), how these specific pathways relate to immunity or exposure to *H. polygyrus* is currently unclear.

Examining immune genes more specifically confirmed known associations between gastrointestinal helminths and immune factors, and revealed others that merit further study. Among previously reported associations was the negative correlation between parasite burdens and the receptor for IL-5, which is the main cytokine involved in the activation of eosinophils, part of the group-2 innate lymphoid cell-driven responses to helminths in the gut (48). Also consistent with previous reports in laboratory models was the negative relationship between *H. polygyrus* and IL-17A. Indeed, this parasite has previously been reported to inhibit IL-17 production in the gut mucosa (72), and more recently, that this interaction may be mediated by a subset of gut-resident eosinophils that suppress IL-17 (47, 73–75). Furthermore, interactions between *H. polygyrus*, Th17, and regulatory T cell responses have been reported to interact with the gut microbiota and be genotype dependent. Indeed, a susceptible mouse strain has elevated IL-17 and increased proportions of *Lactobacilli* whereas in a resistant mouse strain, *H. polygyrus* has no effect on *Lactobacillus* abundance (76). In wild wood mice, these interactions are likely to be driven, in part, by seasonal changes in diet (77), which is consistent with three-way interactions observed between hosts, helminths, and their microbiota in wild seabirds (78). We also detected strong positive associations between *H. polygyrus* burdens and TAB 2 expression, which is reported to activate IL-1 *via* JNK and NF-κB (79). This is consistent with their positive correlation with the reactive oxygen species-expressing NOD-like receptor signaling pathway (80) we identified among WGCNA clusters. This suggests either an inflammatory response to *H. polygyrus* or to the damage it may cause in the gut (81), or that immune systems already skewed toward inflammation are less able to control parasitic helminths. While we did not detect clear signatures of regulatory T cell activation in the spleen, two genes potentially involved in immune regulation correlated strongly with parasite burdens: IL17RA, as mentioned above, and ACKR3, a chemokine scavenger (82) that is widely expressed in the hematopoietic system, heart, vascular endothelial cells, bone, kidney, and brain, and that is reported to be upregulated in many cancers (83) and also mimicked by a herpesvirus agonist (84, 85).

Interestingly, we did not detect significant associations between worm burdens and cytokines or chemokines, but rather with that of their receptors. Likewise, we might expect GATA-3 to correlate with *H. polygyrus* burdens, since this transcription factor is central to ILC2 initiating Th2 responses to helminths in the gut (48). This suggests that in the spleen associations between infection burdens and RNA expression of cytokine receptor genes are more statistically robust, and thus more functionally interpretable, than those between infection burdens and cytokine messenger RNA.

Having observed no significant difference between male and female mice in their parasite burdens, we also did not detect significant differences in the expression of any of the top predictors of parasite burdens, and host sex only marginally explaining the variation in the expression of ACKR3. More surprising was that a 60-fold reduction in *H. polygyrus* burdens within an individual mouse would have so little effect on the immune pathways that predict their chronic burdens. This may be due to the target of the anthelminthic drugs we used, ivermectin and pyrantel (86). The combination of immune-regulatory predictors of chronic infection burdens, the mode of action of commonly used anthelminthics, and the ensuing lack of immune response to drug-induced worm death may explain the high reinfection rates post drug-removal (3–5).

Finally, while wood mice infected with high worm burdens may be more tolerant to infection [i.e., the ability of hosts to minimize adverse fitness consequences of increasing parasite burdens (87)], our study could not address this question satisfactorily because we did not collect data to assess fitness-relevant effects of infection nor of drug treatment in the wood mice. While recent studies suggest a complex relationship between protective immunity, resource limitation, and immunopathology (88, 89), how organisms balance the benefits of eliminating helminth infection and the costs of mounting the anthelminthic immune responses to do so is poorly understood. A further limitation of this study is its reliance on the transcriptome to assess immune responses: in the future, it will be important not only to validate our findings in additional wood mouse populations, e.g., using quantitative RT-PCR, but also to integrate protein, cellular, and metabolomic data alongside transcriptomic data (90) to reduce the risk of overlooking important processes that vary in their post-transcriptional regulation.

In conclusion, we have generated the first transcriptome for *A. sylvaticus* and identified a number of transcriptional predictors of chronic infection chronic infection burdens by *H. polygyrus* that include previously known immune pathways as well as novel candidates which merit further investigation. Notably, resolving the causal relationships between hosts, parasitic helminths and microbiota in the maintenance of immune homeostasis even in the face of drug-induced parasite removal, merits further attention. By combining two distinct wood mouse populations, integrating two different sequencing platforms, and applying machine-learning-based cross-validation procedures to map transcript expression levels to parasite burdens, we have sought to maximize the generalizability and functional relevance of our analysis of the wood mouse immune system. In the future, longer term studies and the integration of multiple biological levels, from genomes to cells, should help further our understanding of how the immune system maintains the health of the organism in its natural habitat.

## Ethics Statement

All procedures on animals were approved by the University of Glasgow ethics committee and the UK Home Office (PPL60/4572) and conducted in accordance with the Animals (Scientific Procedures) Act 1986.

## Author Contributions

SB and AP conceived, designed, and secured funding for the study, ran the field sites, participated in the field and lab work, and wrote the manuscript. SB performed all statistics and machine learning analyses. WL performed the digital transcriptomics and helped write the manuscript. GH performed all assembly and annotation of the transcriptomes. EK extracted and quality-checked all RNA samples. MC and ER participated in the field work and analyzed the parasite samples.

## Conflict of Interest Statement

The authors declare that the research was conducted in the absence of any commercial or financial relationships that could be construed as a potential conflict of interest.
