# Supplementary material for: The Immune and Non-Immune Pathways That Drive Chronic Gastrointestinal Helminth Burdens in the Wild
Source: Front Immunol. 2018 Feb 5;9:56. doi: 10.3389/fimmu.2018.00056 (PMC5807686; doi:10.3389/fimmu.2018.00056)
Supplement: Supplementary file 1 [file presentation_1.PDF]

Supplementary information

Supplementary figures

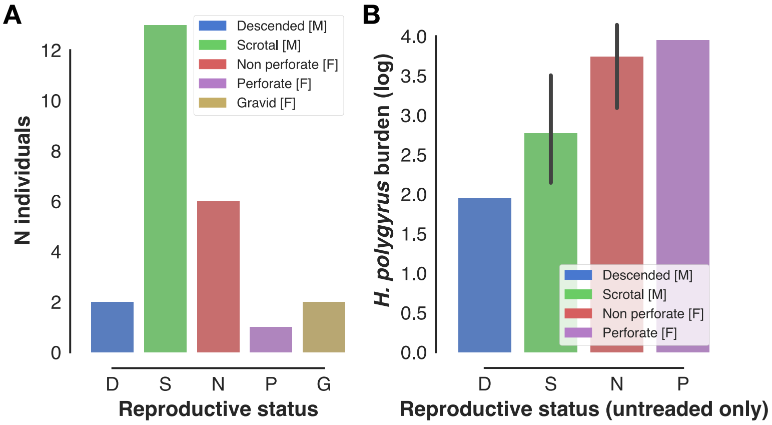

Figure S1: ***A. sylvaticus* distribution between reproductive categories**

Number of male [M] and female [F] adult *A. sylvaticus* and their reproductive status at experiment endpoint.

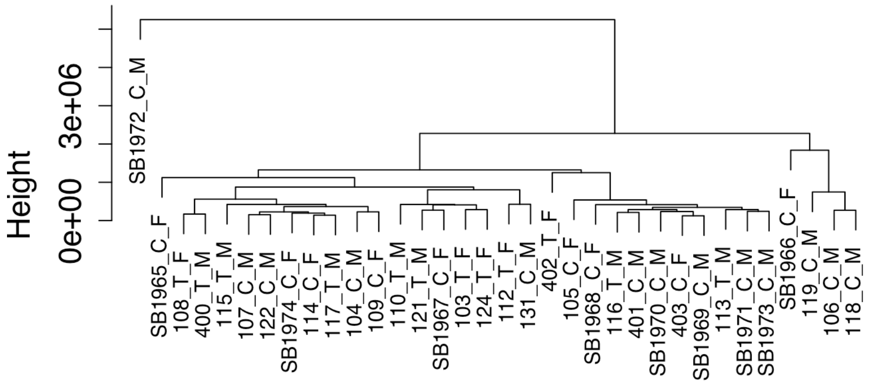

Figure S2: WGCNA sample clustering

Clustering of weighted transcript co-expression networks in the spleen by host sex, treatment, and population of origin. Very weak clustering by either parameter suggests they explain little of the inter-individual variability in gene expression profiles in these populations.

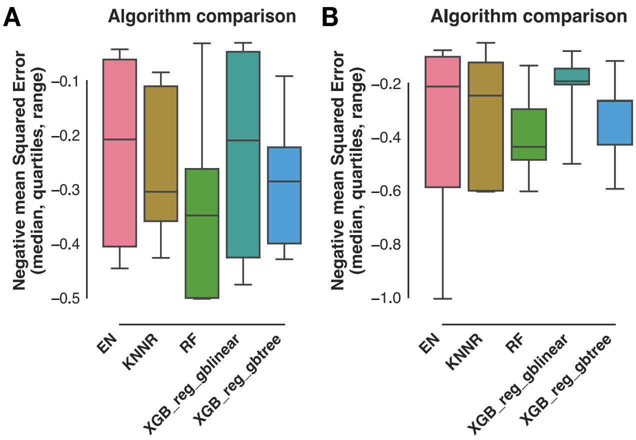

Figure S3: Comparison of machine learning algorithms

Boxplots show the baseline median, quartiles and range of negative mean square errors for 6 algorithms from 10-fold cross validation of non-drug-treated mice (N = 24) for predicting *H. polygyrus* infection burdens. A, mapping WGCNA clusters of whole transcriptome to log-transformed *H. polygyrus* worm counts. B, mapping immune genes to log-transformed *H. polygyrus* worm counts. EN, Elastic Net; KNNR, k-Nearest Neighbours for Regression; RF, Random Forest regressor; XGB, eXtreme Gradient Boosting regressor using either a linear (GLM) booster, or a tree booster.

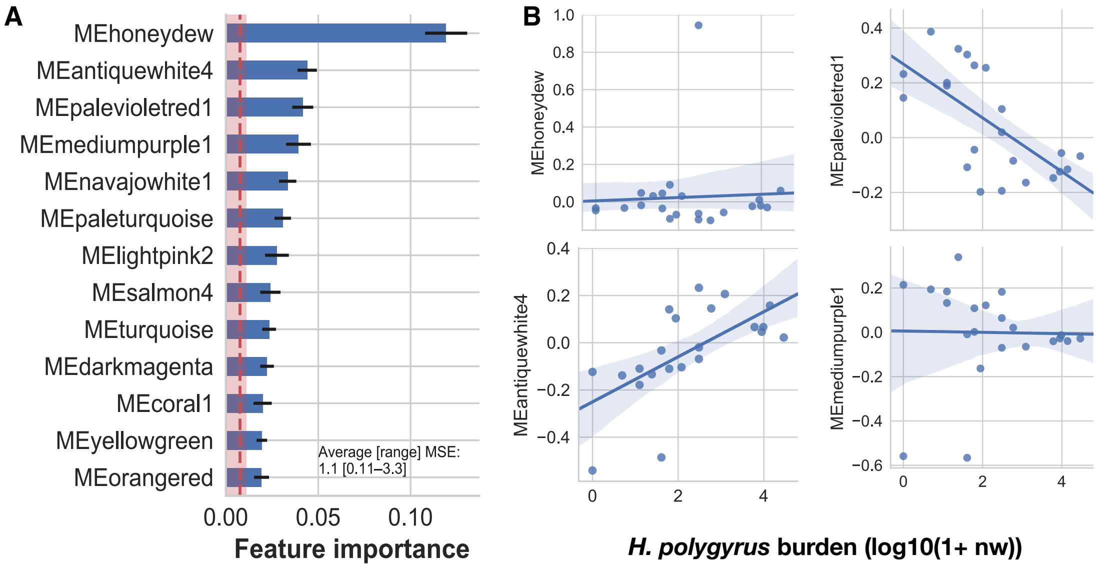

Figure S4: XGBoost WGCNA importances and regression between top features and *H. polygyrus* worm counts

Gradient boosted regression models were used to map WGCNA clusters to *H. polygyrus* worm burdens in untreated mice. A, rank of the importance of each cluster in predicting worm burden. On average, 50 XGBoost cross-validated models built on 10 random 75% / 25% train/test splits predicted worm burdens with an MSE of 1.1 ranging [0.11–3.3]. B, among the top features, only those correlating with worm burden at $p \leq 0.01$ are shown. Horizontal bars represent means and associated errors of the feature importances generated from models trained on and testing against 10 repeated 75% / 25% random splits of the full dataset. In regression plots, each point represents a wood mouse, the solid line is the regression line and the shaded areas represent the corresponding 95% bootstrapped confidence intervals.

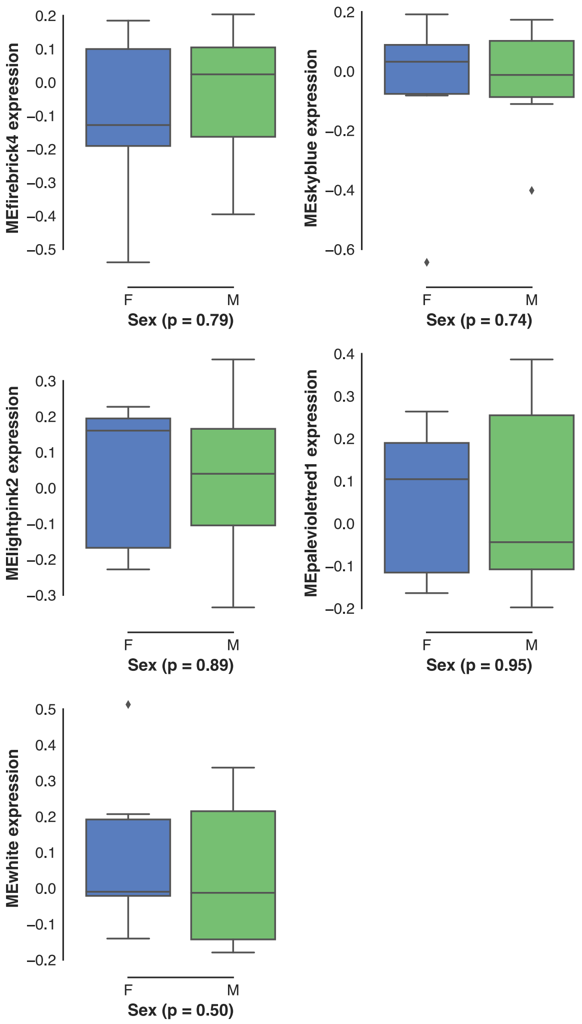

Figure S5: Effects of host sex on parasite burden-associated WGCNA clusters

No statistically significant effects of host sex were detected among the WGCNA clusters that best predicted *H. polygyrus* burdens in untreated mice. Each plot represents transcript counts scaled to unit variance but not centred. Horizontal bars represent the median, boxes represent the interquartile range, whiskers the range, and diamonds transcript counts that lay beyond 1.5x of the interquartile range.

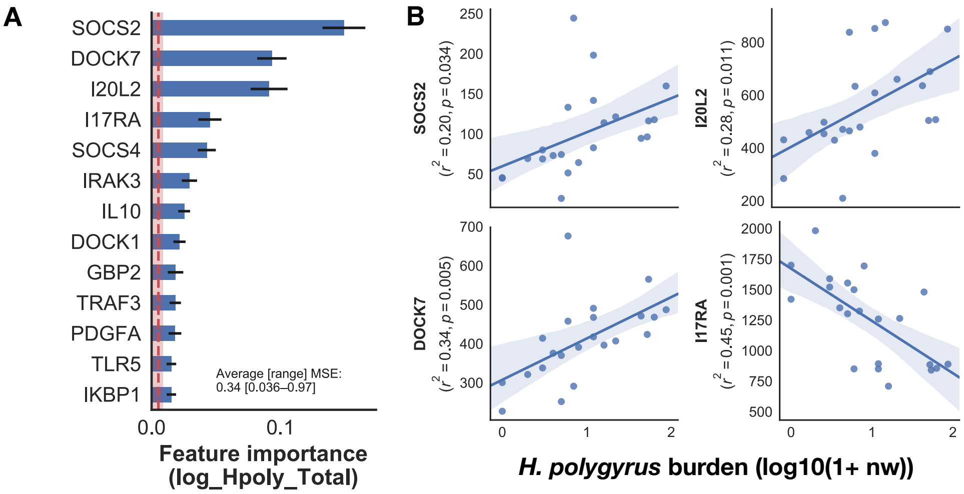

Figure S6: XGBoost immune gene importances and regression between top features and *H. polygyrus* worm counts

Gradient boosted regression models were used to map transcript expression levels to *H. polygyrus* worm burdens in untreated mice. A, rank of the importance of each gene in predicting worm burden. On average, 50 XGBoost cross-validated models predicted worm burdens with an MSE of 0.34 ranging [0.036–0.97]. B, among the top features, only those correlating with worm burden at $p \leq 0.01$ are shown. Horizontal bars represent means and associated errors of the feature importances generated from models trained on and testing against 10 repeated 75% / 25% random splits of the full dataset. In regression plots, each point represents a wood mouse, the solid line is the regression line and the shaded areas represent the corresponding 95% bootstrapped confidence intervals.

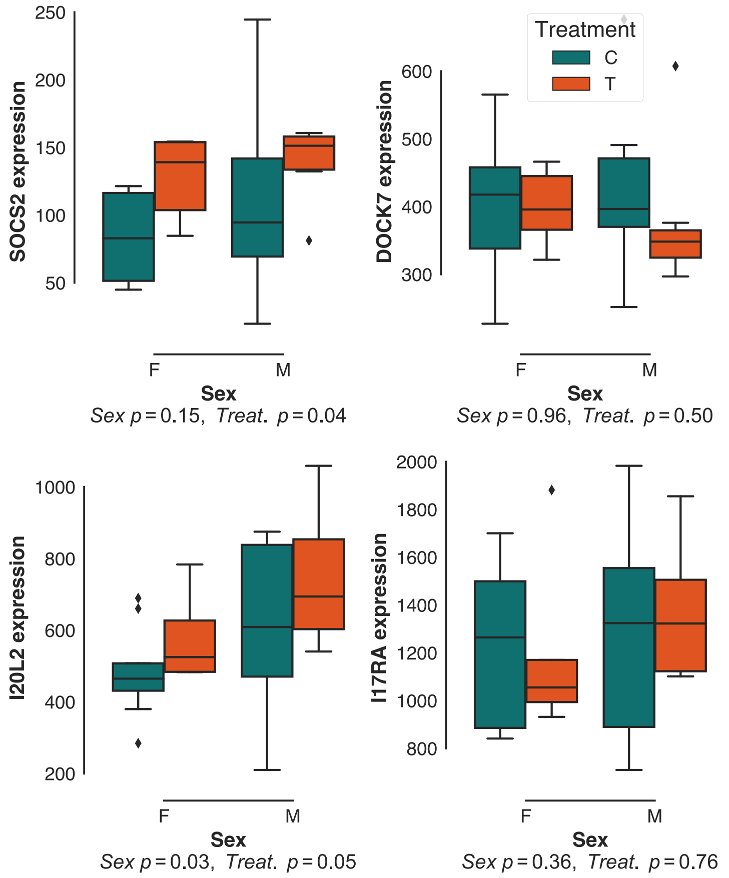

Figure S7: Effects of sex and treatment on top XGBoost predictors of *H. polygyrus* burden

No statistically significant effects of host sex and drug treatment were detected among the best predictors of *H. polygyrus* burdens identified in untreated mice only by XGBoost (at $p \leq 0.01$). Each plot represents transcript counts scaled to unit variance but not centred. Horizontal bars represent the median, boxes represent the interquartile range, whiskers the range, and diamonds transcript counts that lay beyond 1.5x of the interquartile range.

Supplementary tables

Cluster	KEGG pathway description	Adjusted p value	Gene names
**firebrick4**	**Terpenoid backbone biosynthesis**	**0.0482**	**FACE1**
**green4**	Pantothenate and CoA biosynthesis	0.0247	VNN1
**lavenderblush1**	Adherens junction	0.0394	PTPRJ
**lightblue3**	Measles	0.0004	STAT2, IFIH1, DSRAD
**lightblue3**	Influenza A	0.0004	STAT2, IFIH1, DSRAD
**lightblue3**	Hepatitis B	0.0114	STAT2, IFIH1
**lightblue3**	Herpes simplex infection	0.0140	STAT2, IFIH1
**mediumpurple**	Shigellosis	0.0309	WASF2
**mediumpurple**	Adherens junction	0.0309	WASF2
**mediumpurple**	Bacterial invasion of epithelial cells	0.0309	WASF2
**mediumpurple**	Salmonella infection	0.0309	WASF2
**mediumpurple**	Fc gamma R-mediated phagocytosis	0.0309	WASF2
**mediumpurple**	Phosphatidylinositol signaling system	0.0309	VIP1
**mediumpurple**	Choline metabolism in cancer	0.0309	WASF2
**mediumpurple2**	Inositol phosphate metabolism	0.0020	PLCB4, OCRL, IMPA3
**mediumpurple2**	Phosphatidylinositol signaling system	0.0025	PLCB4, OCRL, IMPA3
**plum4**	Terpenoid backbone biosynthesis	0.0181	PCYOX
**plum4**	AMPK signaling pathway	0.0411	CAB39
**plum4**	mTOR signaling pathway	0.0411	CAB39
**salmon**	Drug metabolism - cytochrome P450	0.0355	FMO3, FMO1, ADH7
**skyblue**	**NOD-like receptor signalling pathway**	**0.0005**	**XIAP, TRPM7, MFN2, DHX33**
**skyblue2**	Fc gamma R-mediated phagocytosis	0.0427	MYO10, GAB2
**skyblue4**	Inositol phosphate metabolism	0.0027	MTMR6, MTM1
**skyblue4**	Phosphatidylinositol signaling system	0.0027	MTMR6, MTM1
**skyblue4**	Glycosylphosphatidylinositol (GPI)-anchor biosynthesis	0.0228	PIGH
**violet**	Prostate cancer	0.0183	SOS1, PDPK1, CREB1
**violet**	Neurotrophin signaling pathway	0.0183	SOS1, PDPK1, GAK
**violet**	AMPK signaling pathway	0.0183	PDPK1, CREB1, ACACA
**violet**	Insulin signaling pathway	0.0202	SOS1, PDPK1, ACACA

Table S1: KEGG pathways positively associated with *H. polygyrus* burden

Pathways and included genes positively associated with parasite burdens. Bold underlined rows were significantly correlated with worm burden (in Fig 2B, Cluster 1 = firebrick4, Cluster 7 = skyblue).

Cluster	KEGG pathway description	Adjusted p value	Gene names
**darkolive-green2**	Glycine, serine and threonine metabolism	0.0462	SARDH
**darkolive-green2**	Thyroid hormone signaling pathway	0.0462	GATA4
**darkolive-green2**	Cellular senescence	0.0462	GATA4
**darkolive-green2**	cGMP-PKG signaling pathway	0.0462	GATA4
**darkolive-green2**	Tight junction	0.0462	GATA4
**honeydew**	Tight junction	0.0010	MYH3, MYH2, MYH13
**honeydew**	Phospholipase D signaling pathway	0.0154	GRM8, DGKI
**lightpink2**	**Apoptosis**	**0.0168**	**DFFB**
**orangered1**	Autophagy - other	0.0088	ATG2A
**orangered1**	Autophagy - animal	0.0176	ATG2A
**orangered4**	Protein digestion and absorption	0.0005	TRY3, CTRL, CTRB1
**orangered4**	Pancreatic secretion	0.0005	TRY3, CTRL, CTRB1
**palevioletred1**	**C2H2 zinc finger domain binding**	**0.0005**	**EHMT1, PAX2, WT1**
**paleturquoise**	Hematopoietic cell lineage	0.0416	TRFR, GPV, GP1BA
**paleturquoise**	Platelet activation	0.0416	P2RX1, GPV, GP1BA
**pink3**	Cysteine and methionine metabolism	0.0247	SAHH
**pink3**	Spliceosome	0.0365	DDX23
**salmon4**	Neuroactive ligand-receptor interaction	0.0083	VIPR1, GRPR, GLP1R, CCKAR
**steelblue**	Phosphatidylinositol signaling system	0.0008	PLCB2, P85B, ITPR3, DGKZ, DGKA
**steelblue**	Phospholipase D signaling pathway	0.0021	PLCB2, P85B, DGKZ, DGKA, ADCY3
**steelblue**	Gastric acid secretion	0.0021	PLCB2, ITPR3, HRH2, ADCY3
**steelblue**	Insulin secretion	0.0026	STX1A, PLCB2, ITPR3, ADCY3
**steelblue**	Inflammatory mediator regula-tion of TRP channels	0.0030	PLCB2, P85B, ITPR3, ADCY3
**steelblue**	Estrogen signaling pathway	0.0030	PLCB2, P85B, ITPR3, ADCY3
**steelblue**	Rap1 signaling pathway	0.0038	PLCB2, P85B, EFNA2, AFAD, ADCY3
**steelblue**	Cholinergic synapse	0.0038	PLCB2, P85B, ITPR3, ADCY3
**steelblue**	Platelet activation	0.0049	PLCB2, P85B, ITPR3, ADCY3
**steelblue**	Apelin signaling pathway	0.0066	PLCB2, MEF2D, ITPR3, ADCY3
**steelblue**	cGMP-PKG signaling pathway	0.0115	PLCB2, MEF2D, ITPR3, ADCY3
**steelblue**	Thyroid hormone synthesis	0.0121	PLCB2, ITPR3, ADCY3

**steelblue**	Calcium signaling pathway	0.0140	PLCB2, ITPR3, HRH2, ADCY3
**steelblue**	Aldosterone synthesis and secretion	0.0140	PLCB2, ITPR3, ADCY3
**steelblue**	Gap junction	0.0160	PLCB2, ITPR3, ADCY3
**steelblue**	Salivary secretion	0.0160	PLCB2, ITPR3, ADCY3
**steelblue**	GnRH signaling pathway	0.0160	PLCB2, ITPR3, ADCY3
**steelblue**	Circadian entrainment	0.0162	PLCB2, ITPR3, ADCY3
**steelblue**	Pancreatic secretion	0.0162	PLCB2, ITPR3, ADCY3
**steelblue**	Choline metabolism in cancer	0.0168	P85B, DGKZ, DGKA
**steelblue**	Glutamatergic synapse	0.0239	PLCB2, ITPR3, ADCY3
**steelblue**	Vascular smooth muscle contraction	0.0270	PLCB2, ITPR3, ADCY3
**steelblue**	Relaxin signaling pathway	0.0316	PLCB2, P85B, ADCY3
**steelblue**	Carbohydrate digestion and absorption	0.0377	PLCB2, P85B
**steelblue**	Retrograde endocannabinoid signaling	0.0417	PLCB2, ITPR3, ADCY3
**steelblue**	Oxytocin signaling pathway	0.0439	PLCB2, ITPR3, ADCY3
**steelblue**	Regulation of lipolysis in adipocytes	0.0499	P85B, ADCY3
**tan4**	Tight junction	0.0234	MYH7
**white**	**ABC transporters**	**0.0337**	**ST14, ABCD4**

Table S2: KEGG pathways negatively associated with *H. polygyrus* burden

Pathways and included genes negatively associated with parasite burdens. Highlighted rows were significantly correlated with worm burden (in Fig. 2B, Cluster 23 = white, Cluster 24 = lightpink2, Cluster 25 = palevioletred1).
